# Visualizing RNA polymers produced by hot wet-dry cycling

**DOI:** 10.1038/s41598-022-14238-2

**Published:** 2022-06-23

**Authors:** Tue Hassenkam, David Deamer

**Affiliations:** 1 Globe Institute, University of Copenhagen, 1350 Copenhagen, Denmark; 2 Department of Biomolecular Engineering, University of California, Santa Cruz, Santa Cruz, CA 95064 USA

**Keywords:** Origin of life, Biochemistry, Nanoscience and technology

## Abstract

It is possible that the transition from abiotic systems to life relied on RNA polymers that served as ribozyme-like catalysts and for storing genetic information. The source of such polymers is uncertain, but previous investigations reported that wet–dry cycles simulating prebiotic hot springs provide sufficient energy to drive condensation reactions of mononucleotides to form oligomers and polymers. The aim of the study reported here was to verify this claim and visualize the products prepared from solutions composed of single mononucleotides and 1:1 mixture of two mononucleotides. Therefore, we designed experiments that allowed comparisons of all such mixtures representing six combinations of the four mononucleotides of RNA. We observed irregular stringy patches and crystal strands when wet-dry cycling was performed at room temperature (20 °C). However, when the same solutions were exposed to wet–dry cycles at 80 °C, we observed what appeared to be true polymers. Their thickness was consistent with RNA-like products composed of covalently bonded monomers, while irregular strings and crystal segments of mononucleotides dried or cycled at room temperature were consistent with structures assembled and stabilized by weak hydrogen bonds. In a few instances we observed rings with short polymer attachments. These observations are consistent with previous claims of polymerization during wet–dry cycling. We conclude that RNA-like polymers and rings could have been synthesized non-enzymatically in freshwater hot springs on the prebiotic Earth with sizes sufficient to fold into ribozymes and genetic molecules required for life to begin.

## Introduction

Although the specific chemical processes and environments that led to the emergence of living systems are unknown, at least two general possibilities have been proposed. One is that life began in the ocean in hydrothermal vents where mineral compartments may have encapsulated and catalyzed chemical reactions which then evolved into a primitive metabolism^1–4^. Polymers such as peptides and oligonucleotides could be generated within the compartments and begin to function as catalysts and carriers of genetic information, setting the stage for a primitive living system^5–7^.

The alternative is that polymers were synthesized by a separate process prior to the advent of cellular life, and these were able to evolve and reproduce in the absence of metabolism. A version of Darwinian evolution could have been initiated at this early stage through direct selection of reproducing polymers in a localized environment^8–10^. The initial selective factors were stability, porosity and ultimately the emergence of molecular systems capable of catalyzed growth, genetically directed polymer synthesis and a primitive metabolism^11^.

This alternative represents a testable hypothesis in which nucleotides and polymers can be synthesized non-enzymatically in freshwater hydrothermal surface environments associated with volcanic activity on a young Earth^12,13^. The hypothesis has undergone extensive testing over the past 15 years. The first experiments were undertaken by Rajamani et al.^14^ and the results were encouraging. Mononucleotides were exposed to laboratory simulations of hydration and dehydration (now referred to as wet-dry cycles) that occur in and around hot springs. The nucleotide mixtures exposed to wet-dry cycles contained polymers that exhibited UV spectra expected if they were composed of nucleotide monomers. Furthermore, they could be end-labeled by ATP-32 in a reaction catalyzed by T-4 kinase. The labeled polymers were visualized by gel electrophoresis and moved in the gel as expected if they were linear polyanions. This observation was confirmed by nanopore analysis. Large numbers of ionic current blockades were detected when the polymers were monitored as they were moved through the hemolysin nanopore by an applied voltage. This was only possible if linear polyanions had been synthesized by the wet-dry cycling. Finally, the presence of phospholipids in the mixture did not inhibit the polymerization and polymeric products could be visualized within self-assembled vesicles when they were stained by intercalating dyes. The experiments were repeated by Da Silva et al*.*^15^ who discovered that polymer yields were markedly increased if ammonium chloride was present in the mixture. DeGuzman et al.^16^ extended the nanopore analysis by comparing the characteristic ionic current blockades of polymers synthesized by wet-dry cycling to those produced by known species of synthetic RNA.

That foundation led to the experiments being reported here. According to the hot spring hypothesis it is essential to have sufficient activation energy available for polymerization to take place. That is, the temperature must be high enough to activate condensation reactions in which mononucleotides can be linked into polymers by ester bond synthesis without the need of a catalytic agent, but not so high that other chemical bonds cross-link potential reactants and cause tars to form^17^. It follows that polymers are very unlikely to be produced if mononucleotides are exposed to wet-dry cycles at 20 °C where activation energy is insufficient. On the other hand, if wet-dry cycles are performed at 80 °C, a temperature range characteristic of volcanic hot springs, sufficient activation energy is available to allow abundant synthesis of polymers resembling nucleic acids^18^.

A preliminary atomic force microscopy (AFM) study reported that products from wet-dry cycling of mixtures mononucleotides could be seen adhering to mica surfaces both as particles and as ring structures 30–40 nm in diameter^19^. In the present study we used AFM to investigate the effect of temperature on the kinds of molecular structures that form on atomically smooth mica surfaces when mononucleotides are exposed to wet-dry cycles at 20 °C and 80 °C. We investigated both single nucleotides and combinations of two mononucleotides in 1:1 mol ratios. Preliminary experiments suggested that mixtures of two mononucleotides capable of Watson–Crick base pairing such as AMP + UMP or GMP + CMP have properties beyond those of individual mononucleotides or mismatched mixtures such as AMP + CMP or GMP + UMP. Therefore, we designed experiments that allowed comparisons of all such mixtures representing six combinations of the four mononucleotides of RNA.

## Results

### Mononucleotides dried at room temperature

We performed a series of controls to make sure that the polymers visible by atomic force microscopy (AFM) were in fact being generated by the wet-dry cycling rather than contamination. One such control was to dry reaction mixtures on a mica surface at room temperature and then search for possible contaminating polymers that resembled known nucleic acid polymers examined by AFM. If obvious polymers were present, it would indicate contamination. We did not observe any polymers in the control experiments but did find other interesting structures that revealed how the mononucleotides interacted with each other and the mica surface at room temperature.

**Figure 1:**
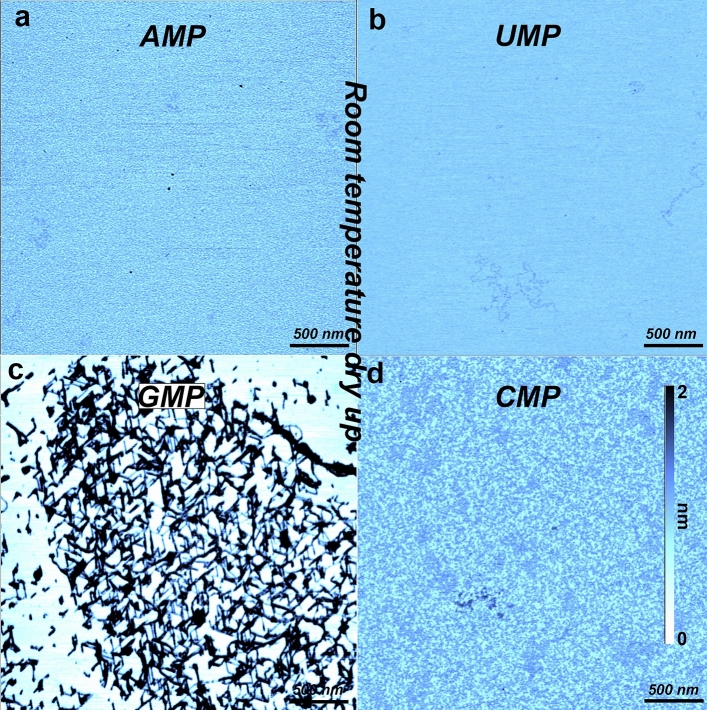
AFM images of 10 mM solutions of mononucleotides dried on mica surfaces. (**a**) Adenosine monophosphate (AMP), (**b**) Uridine monophosphate (UMP), (**c**) Guanosine monophosphate (GMP) The striking rod-like structures that formed from GMP have been previously observed^23^. They are produced when concentrated GMP forms quadruplex structures that stack into long rods which become aligned with the crystalline surface of the mica. (**d**) Cytidine monophosphate (CMP). To make the images comparable the color scale covers the range from 0 to 2 nm for all images.

Figure 1 shows the results from allowing four different nucleotide solutions (10 mM in water, pH 3) to dry at room temperature on a freshly cleaved mica surface. The images show representative AFM scans of the surface after rinsing with a stream of ultrapure water and then dried under a jet of clean nitrogen gas. A variety of structures were visible on the surfaces. The solution of AMP (Fig. 1a), exhibited a mostly homogeneous surface with a few undefined patches that protruded about 0.1 nm from the surrounding surface. The overall roughness of the surface was about 0.3 nm.

Figure 1b shows an AFM scan of a surface on which a solution of UMP was dried at room temperature. Here isolated irregular strings of material were observed. The strings appeared to form single strands that occasionally assembled into loops. The thickness of the strings varied between 0.1 and 0.3 nm.

When a solution of GMP molecules was dried, short rod-like segments with specific orientations related to the crystalline mica surface were present. The thickness of the segments was typically 2 nm but a few were around 1 nm with a characteristic length around 150 nm. In many cases the segments were oriented mainly in three directions giving rise to 60-degree angles between segments.

The surfaces on which a solution of CMP was dried displayed a porous surface with roughness around about 0.3 nm with a few irregular patches that extended 0.6 nm from the deepest point in the image.

### Nucleotide mixtures dried at room temperature

**Figure 2:**
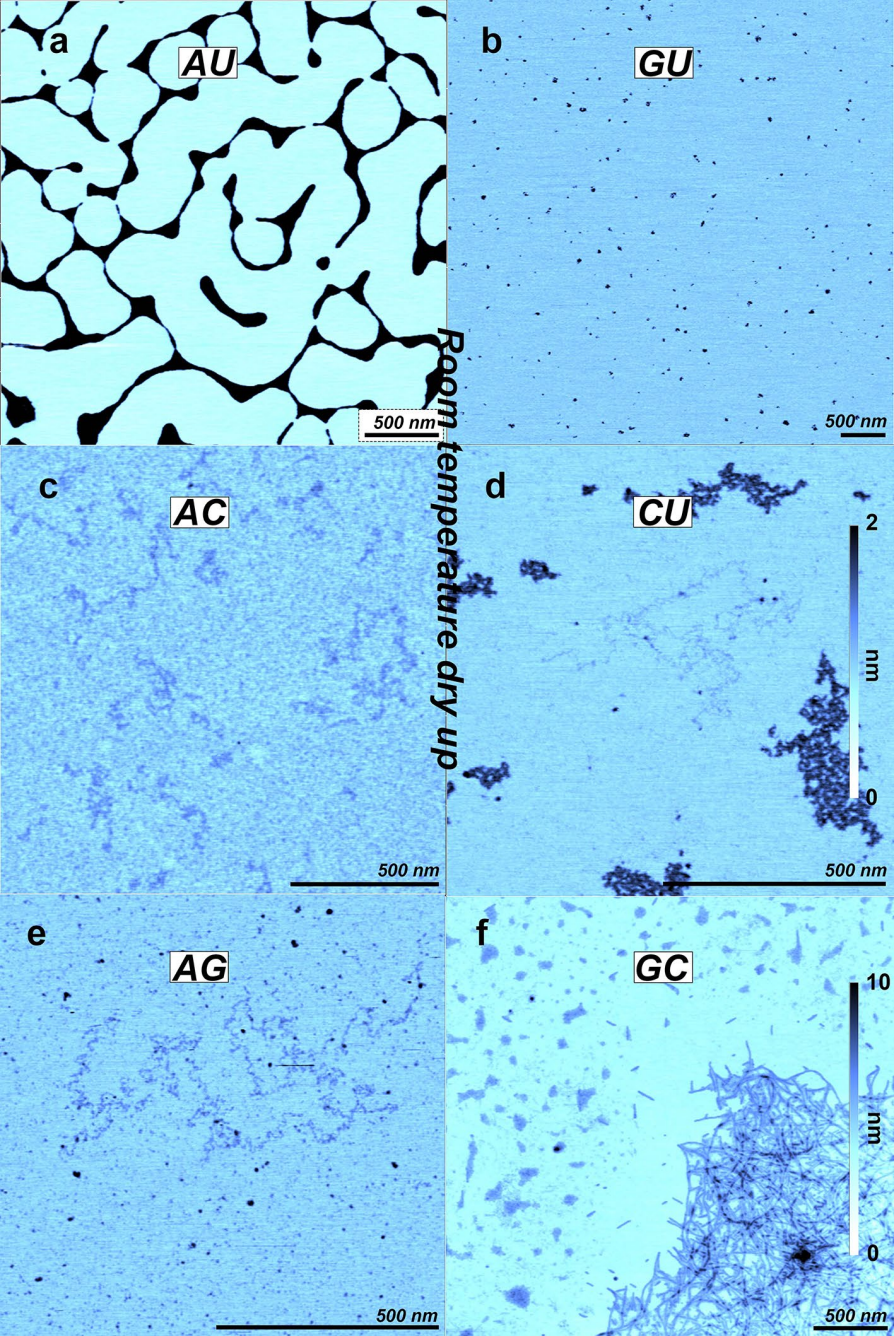
AFM images of 1:1 mixtures of 10 mM mononucleotides dried at room temperature on a mica surface. (**a**) AMP + UMP, (**b**) GMP + UMP, (**c**) AMP + CMP, (**d**) CMP + UMP, (**e**) AMP + GMP, (**f**) GMP + CMP To make the images comparable the color scale covers the range from 0 to 2 nm for (**a**)–(**e**), for (**f**) it covers the range from 0 to 10 nm.

Next, we tested mixtures of two mononucleotides and dried them at room temperature on the mica surfaces (Fig. 2). At this temperature polymers were not expected to form, therefore this experiment also served as a control for contamination. While a variety of structures were observed, those shown here could be reproduced and displayed some regularity or order. The variety of structures is most likely linked to the nonlinear and somewhat chaotic nature of the drying process (see “Methods” for details). Figure 2a shows a mica surface on which a 1:1 mol ratio mixture of AMP and UMP was dried. Rather than patches observed with individual nucleotides, the material formed a dewetting network with varying thicknesses. The minimum thickness of the strings was around 0.9 nm while the larger structures ranged up to 5 nm in thickness. The structures suggest that the mixed monomers were present as films in which molecular aggregates formed the observed network as water evaporated.

Figure 2b shows the surface on which GMP + UMP was dried. This surface shows mostly random particles probably composed of monomer aggregates. The presence of UMP apparently inhibited the formation of larger rod-like structures observed when a GMP solution dried. Irregular patches of stringy material were present on mica surfaces on which the combination AMP + CMP was dried (Fig. 2c). The height of the strings varied from 0.1 to 0.3 nm, and the strings appeared to be on top of a monolayer with a thickness of 0.2–0.3 nm. The mica with the CMP + UMP solution dried on it (Fig. 2d) also displayed very thin 0.1–0.3 nm irregular stringy patches. There were also larger aggregates several hundred nm across and 0.7 nm thick on average. Irregular stringy material was also present on mica surfaces on which the AMP + GMP sample was dried (Fig. 2e). The thickness of these strings was in the range from 0.1 to 0.3 nm, similar to the stringy material seen in the other cases mentioned above. Figure 2f shows the deposits on a surface after drying a GMP + CMP solution. Here there were two different structural packing motifs. The first consisted of long homogenous mostly straight strands around 2 nm thick forming a network with short straight segments of around 100 nm in length dispersed next to the network. The dimensions and the morphology of these segments resembled those found for GMP alone (Fig. 1c). Patches of material of irregular shape and varying size were observed in connections outside the network. These irregular patches came in two thicknesses; around 0.3–1 nm and around 2 nm.

### Mixtures exposed to wet-dry cycles at room temperature

**Figure 3:**
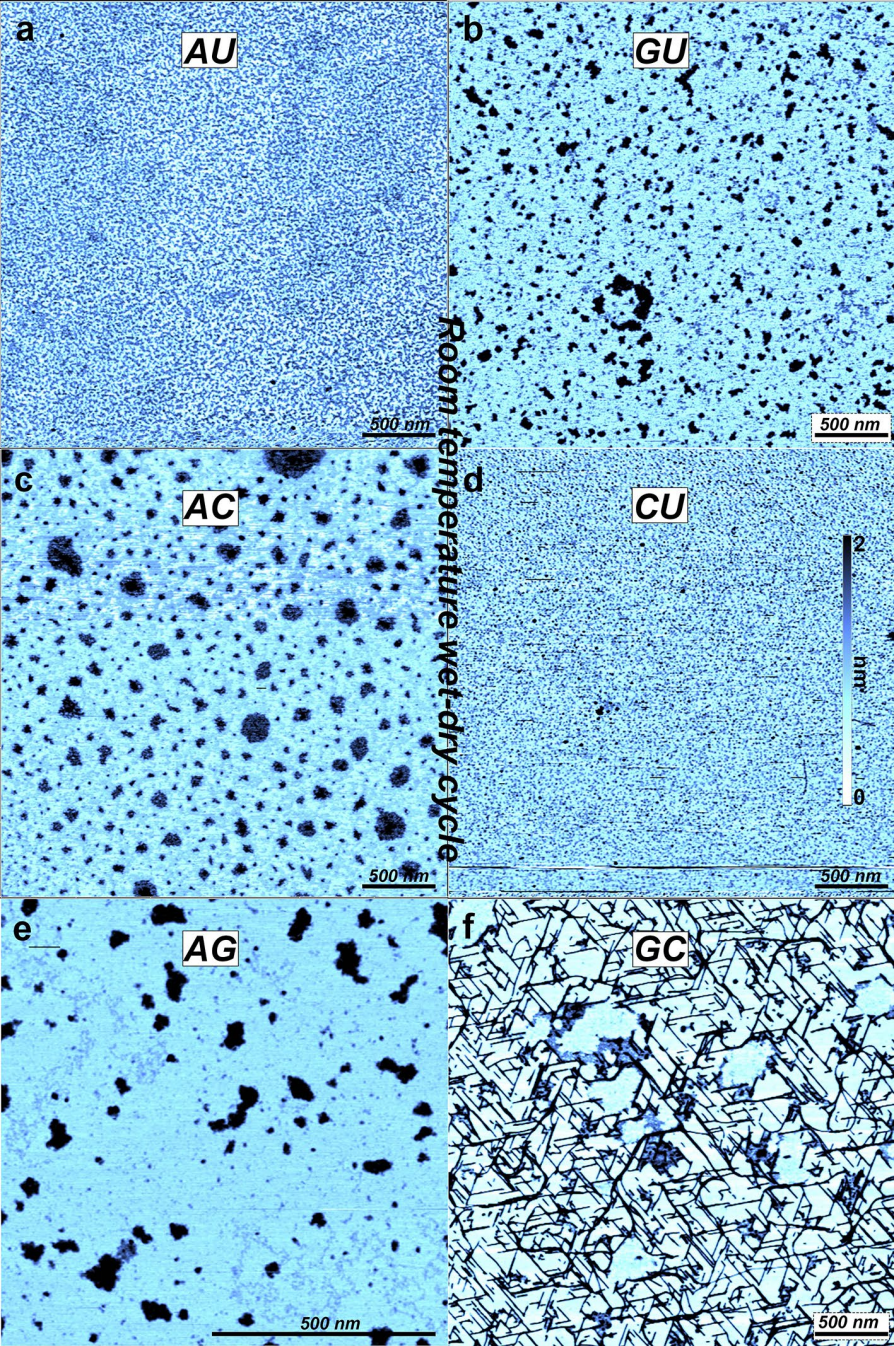
AFM images of 1:1 mixtures of 10 mM mononucleotides which underwent 3 wet-dry cycles on mica surfaces at room temperature. (**a**) AMP + UMP, (**b**) GMP + UMP, (**c**) AMP + CMP, (**d**) CMP + UMP, (**e**) AMP + GMP, (**f**) GMP + CMP. To make the images comparable the color scale covers the range from 0 to 2 nm for all the images.

To test if temperature was critical for polymers to form, we performed controls in which mononucleotide mixtures were exposed to multiple wet-dry cycles at room temperature. Figure 3 shows the result when mixed nucleotide solutions were allowed to go through 3 wet-dry cycles on a clean mica surface at room temperature. In the final step the surface excess material was rinsed with ultra-pure water as done with the previous sets of samples. Assuming that the deepest point in the AFM image is the mica surface, the AMP + UMP mixture (Fig. 3a) seemed to generate a perforated layer on the mica surface with a thickness around 0.9 nm. The GMP + UMP mixture (Fig. 3b) created deposits that could be divided into two different patches, one with an average height around 0.8 nm and a second with an average height around 1.5 nm. The AMP + CMP mixture (Fig. 3c) also created irregular patches. The average thickness of these patches was around 1.1 nm. The CMP + UMP mixture (Fig. 3d) did not give rise to any noticeable organization of the molecules. The roughness of the surface was around 0.35 nm. Figure 3e shows structures from the AMP + GMP experiment. Here there were two different packing motifs: large amorphous patches and irregular strings between 0.15 and 0.3 nm thickness. The GMP + CMP structures shown in Fig. 3f have features similar to the GMP + CMP and GMP taken to dryness just once, but the 2 nm thick crystalline segments appear to be much longer and the orientation of the crystals was more clearly guided by the mica crystalline surface.

### Nucleotide mixtures cycled at 80 °C

**Figure 4:**
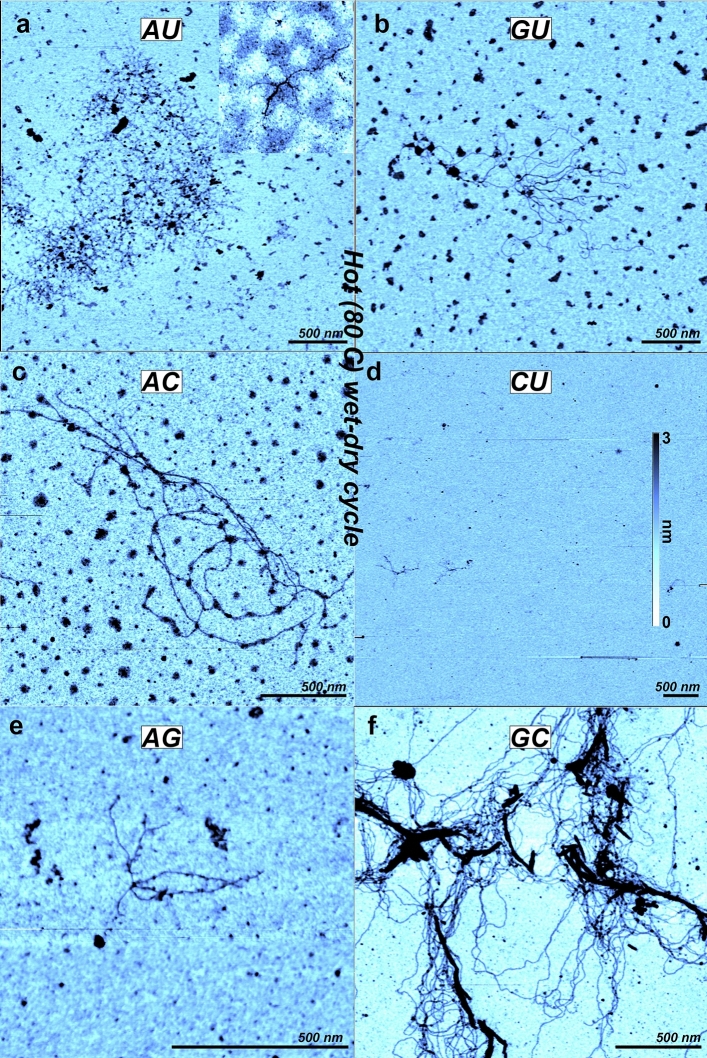
AFM images of 1:1 mixtures of 10 mM mononucleotides exposed to 3 wet-dry cycles on mica surfaces at 80 °C. (**a**) AMP + UMP. Two types of polymer structures are shown, one as an inset at the same magnification. (**b**) GMP + UMP, (**c**) AMP + CMP, (**d**) CMP + UMP, (**e**) AMP + GMP, (**f**) GMP + CMP. To make the images comparable, the color scale covers the range from 0 to 2 nm for all the images.

**Figure 5:**
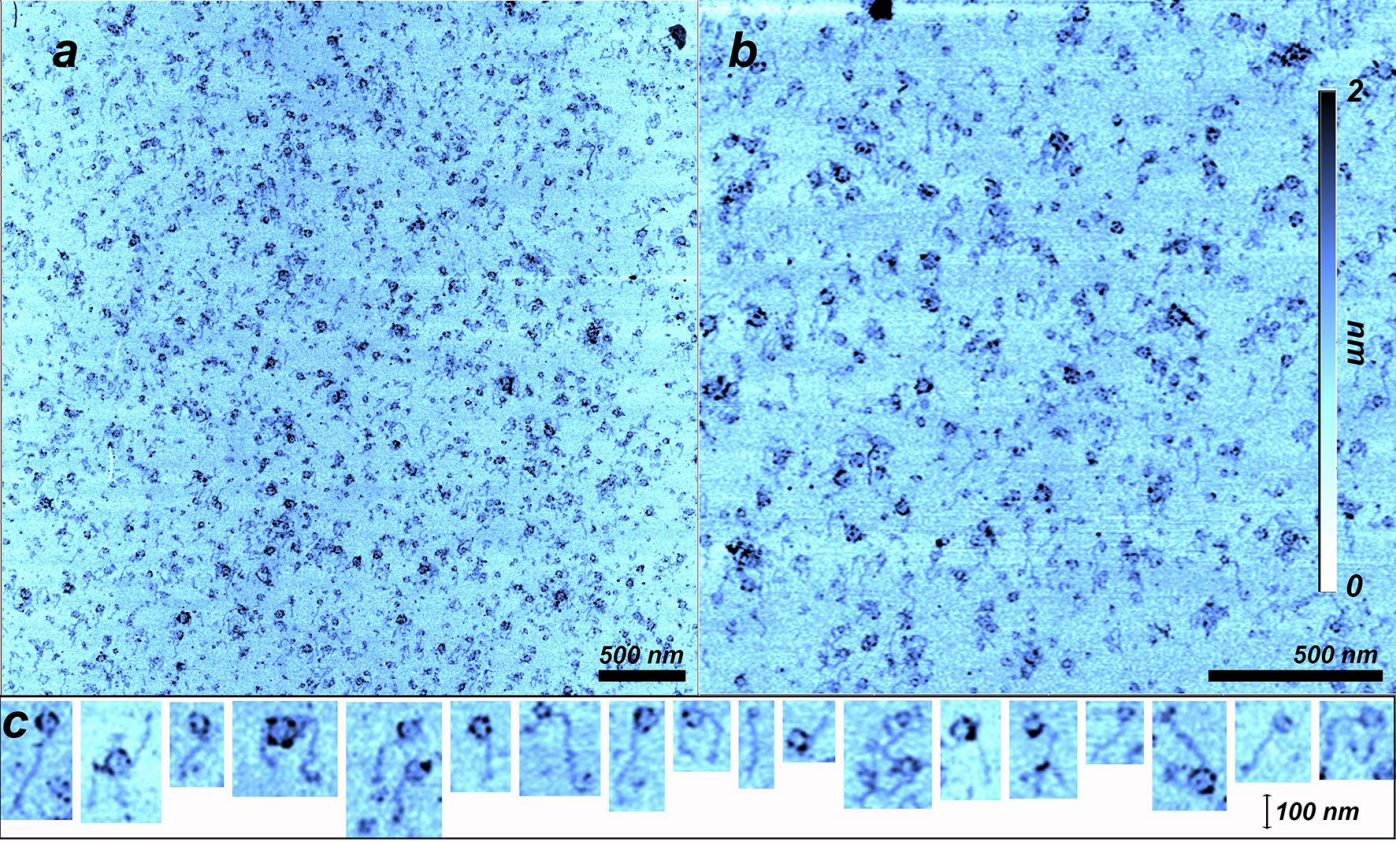
AFM images of rings produced by 80 °C wet-dry cycling of a 1:1 10 mM AMP + UMP solution on a mica surface from two different spots on the same surface. (**a**) 4 µm × 4 µm, (**b**) 2 µm × 2 µm. The color scale covers the range from 0 to 2 nm for all the images. (**c**) Zoom in on structures from (**a**) and (**b**) showing various examples of rings with associated or attached polymers.

We repeated the experiments with the same sets of nucleotides but on a mica surface heated and maintained at 80 °C. The samples were exposed to three wet-dry cycles, and the initial drying process occurred in just a few minutes. The dried samples remained at 80 °C for 30 min before being rehydrated with ultra-pure water. In the final step the surface was rinsed with ultra-pure water as done with the room temperature samples. For all of the mixtures there was clear evidence that apparent polymers had been synthesized and adhered to the mica surface (Fig. 4). Although there was considerable variability from sample to sample, the images were chosen to reflect the range of sizes found in most cases. In some samples ring structures and short polymers a few hundred nanometers in contour length were observed (Figs. 4a and 5). In other samples huge tangles of polymers with contour lengths stretching several micrometers covered the surface, like those observed with AMP + UMP (Fig. 4a), GMP + UMP (Fig. 4b), AMP + CMP (Fig. 4c) and GMP + CMP (Fig. 4f). This corresponds to linking of thousands of nucleotides. In the GMP + UMP case (Fig. 4b) we can track an isolated polymer that is at least 800 nm long, which would then be equivalent to > 2000 nucleotides, assuming 0.34 nm per nucleotide. The density of polymers and the size of the tangles varied across the different nucleotide pairs. It is difficult to give an exact number based on the AFM images alone. But in one end, the GMP + CMP the surface was covered with polymers tangles that extended well beyond several AFM images, in the other end we found few and short polymers in the AMP + GMP and CMP + UMP case. The polymers were in all cases homogenous and regular with thickness around 0.5 or 1 nm for AMP + UMP, 0.7 nm for GMP + UMP, 1 nm for GMP + CMP, 0.5 nm for CMP + UMP, 0.9 nm for AMP + GMP and 0.9 nm for GMP + CMP. In some cases, the polymers combined for a short stretch with the thickness expected for stacked polymers. Other structures were also observed, including irregular small patches of material about 100 nm across and 1–2 nm thick for the GMP + UMP solution (Fig. 4b) and the AMP + CMP solution (Fig. 4c). Semi-crystalline structures are mixed with the tangled polymers of the GMP + CMP products (Fig. 4f). The thickness of these was around 2.5 and up to 5.5 nm, with a minimum thickness of 2 nm.

### Rings from AMP + UMP wet-dry hot cycles

Figure 5 shows a case where extensive ring formation was observed. While we often observed ring formation for AMP + UMP and GMP + CMP samples, there would be instances where very few rings were observed such as the samples shown in Fig. 4a, f. In Fig. 5 the rings ranged from 8 to 40 nm with an average of 30 nm in diameter. The rings were found in a large area that extended well beyond the field shown in the AFM images. There were close to 1000 rings just in Fig. 5a. Many of the rings had polymers about 100 nm in length attached to them, the insets below Fig. 5a and b show 18 examples of rings with such attached polymers. The minimum thickness of the rings was around 0.5 nm, but they were sometimes double or in rare cases triple that. The thickness of the small attached polymers ranged between 0.3 and 0.5 nm.

## Discussion

What properties would we expect to see when solutions of nucleotide monomers are dried on a mica surface? Because the surface of the mica was rinsed with a stream of water, most of the monomers should be washed away but some would adhere individually or in clusters either by Van der Waals forces, by hydrogen bonding or by ionic interactions with the mica surface^20^. The length and width of a single nucleotide molecule is about 0.5 nm. If the molecule is adhering to a mica surface with the planar part parallel to the surface it should be at least 0.3 nm thick. The molecules could also be standing upright on the surface, and a dense monolayer would then be around 0.5 nm thick. If a monolayer were covering the mica surface, we would expect it to have a roughness that reflects the dimensions of the molecules. Even though the general morphology of the surfaces shown for AMP and CMP (Fig. 1a,d) is slightly different, the roughness of 0.3 nm is about the same. When compared with that of clean mica (0.08 nm), the roughness agrees with the size of the tetrahedral phosphate. The patches that rise above the surrounding surface of 0.4 nm AMP and 0.6 nm for CMP, could then be different packing motifs, with molecules either stacked on top of each other or standing upright.

For UMP we observed the 0.3 nm thickness for portions of the irregular stringy structures and 0.1 nm for other portions. This is consistent with loosely hydrogen bonded monomers forming long irregular chains of molecules which are 0.3 nm thick with 0.1 nm spots indicating gaps between molecules. Because UMP has been demonstrated to be capable of non-canonical base pairing^21^ it is possible that hydrogen bonding helped to stabilize the string-like structures that assembled when UMP solutions were dried.

For GMP the structures are consistent with GMP molecules forming quadruplexes that stack in hydrogen bonded rod-like crystals^22^. GMP is unique among mononucleotides in its ability to assemble into stable quadruplexes. The reason is that each guanine can form two hydrogen bonds with a neighboring guanine, resulting in a cyclic quartet stabilized by eight hydrogen bonds. Further stabilization occurs if a monovalent cation is present in the solution that coordinates with the oxygen in each guanine. The quadruplexes can then form linear stacks stabilized by π–π interactions between the planar guanine quartets. These structures have been reported earlier by others^23^ and are the clearest evidence that aggregates of mononucleotides can resist rinsing with water and remain adhering to the mica surface.

Several of the nucleotides mixed in different pairs and dried once or multiple times at room temperature form stringy structures 0.1–0.3 nm thick. We interpret these as loosely hydrogen- bonded monomers that aggregate as they become concentrated during drying. We also see the hydrogen-bonded rod-like structures formed by GMP + CMP mixtures and what appears to be a dense monolayer forming in dried AMP + CMP samples. The same sets of nucleotides either dried once or several times should in principle give rise to similar features, since they both involve simple evaporation on a mica surface. This is true for the AMP + GMP and GMP + CMP mixtures, but the AMP + UMP, GMP + UMP, AMP + CMP and CMP + UMP mixtures dried three times have a different appearance to the same samples taken to dryness just once. One reason could be that the structures are sensitive to the rinsing procedure. The cycles of drying and rehydration may also shift the interactions from a molecule–molecule interaction toward a molecule-surface interaction that allows more molecules to adsorb to the surface. This would be consistent with the apparent monolayer generated by wet-dry cycling of AMP + UMP and CMP + UMP having more material adhering to the surface compared to AMP + CMP and GMP + UMP. The crystalline structures of cycled GMP + CMP also appear to be more strongly bound to the surface compared to the GMP + CMP sample dried just once, and the orientation of the rods is more clearly guided by the crystalline structure of the mica surface, consistent with observations of similar systems in litterature^24^.

The samples generated by three wet-dry cycles at 80 °C have several fundamentally different features from those dried at 20 °C. Only in these samples do we observe what appear to be true polymers. Compared to the irregular strings found in several of the room temperature samples (Figs. 1b, 2), the polymers are thicker, they are continuous, lack kinks, and do not go below 0.5 nm in thickness. The thickness has discrete values, consistent with bundles of polymers having the same diameter. While the thin, irregular strands are most likely linear aggregates of hydrogen-bonded mononucleotides, those produced by hot, wet-dry cycles are best explained as nucleotides linked by ester bonds into RNA-like polymers in which the continuous phosphate-ribose backbone covalently bonded nucleobases ensures that the polymers in AFM images are never thinner than 0.5 nm. This is in contrast to the strings where the 0.1 nm thickness can only be seen by AFM if the phosphate groups are at least 1 nm apart (see “Methods” for details).

The difference between the two types of structures—thin irregular strings that form at room temperature compared to thicker polymers produced by hot wet-dry cycles—is consistent with the difference in temperature during drying. The ester bonds forming non-enzymatically between the sugar and phosphate require activation energy delivered by the elevated temperature. Although hydrogen bonds and Van der Waals interactions between monomers are stable enough at room temperature to generate long irregular strings and the crystals of GMP quadruplexes, these weaker bonds are sensitive to elevated temperatures that cause the structures to break down. This is presumably why the thin irregular structures are not observed in the high temperature samples. However, the fact that they do occur at 20 °C suggests that hydrogen bonding can impose linear order on the nucleotides at lower temperatures. In a sense, these are pre-polymers as suggested by Himbert et al.^25,26^ that make it more likely for neighboring nucleotides to undergo condensation reactions thus forming true polymers. This agrees well with the polymers that seem to be intimately connected to crystalline structures seen in Fig. 4f.

The synthesis of rings and long strands of nucleic acids by such a simple process as wet-dry cycling is surprising. One might expect that long strands of polymerized nucleotides linked by ester bonds should rapidly hydrolyze in an acidic solution at elevated temperatures. How, then, could strands hundreds and even thousands of nucleotides in length survive multiple cycles of wetting and drying? This question has been explored both in terms of thermodynamics and kinetics by Ross and Deamer^18^ who reported that low water activity and highly concentrated reactants provide a source of chemical potential that can drive rapid polymerization of mononucleotides. Furthermore, because the rate of synthesis far exceeds the rate of hydrolysis, polymers will accumulate in a kinetic trap.

Another concern is that polymers of this length might be nucleic acid contaminants already present in the solutions. The commercial nucleotides are particularly suspect in this regard because they are not synthesized chemically but instead are isolated from biological sources. We addressed this concern in several ways. First, laboratory water was eliminated as a source of contaminants because no polymers were ever detected when water alone was dried on mica surfaces. Second, the nucleotide solutions were filtered through 3 kD cutoff filters that removed polymers longer than ~ 10 nucleotides. Finally, if the polymers were contaminants they should adhere to the mica surfaces exposed to nucleotide solutions in the absence of hot cycling. However, ring structures and robust tangles of polymers were only observed when nucleotide mixtures were exposed to wet-dry cycling at 80 °C.

Here we should note that each AFM image only represents a very small portion of the whole sample area. The assumption is that imaging a series of different random spots on the surface should be representative of what can be found on the surface. We designed an experimental protocol to address such issues. First, at least 5 out of 9 AFM images in each of the hot wet-dry cycled samples displayed polymers, and there were no polymers in any of the 9 images in each of the control samples. We can calculate the likelihood of getting a false positive if we assume that contaminating polymers are present. There could be a few polymers which were not seen in the controls but somehow were observed in many images from the hot wet-dry samples, or there could be abundant contaminant polymers which somehow escaped the controls. By combining these two scenarios it turns out that the likelihood of getting a false positive peak at 0.3%. In other words, we are 99.7% confident that the wet-dry cycles are synthesizing true polymers (see supplemental note for details).

If the wet-dry polymers resemble RNA they should have dimensions similar to known RNA molecules in AFM images. To this end, we imaged viroid RNA deposited on mica. We do not claim that what we observe in our images has anything to do with viroids, but the dimensions of the viroids are clearly comparable to the dimensions of our polymers. Viroids are the smallest known infective agents and cause a variety of plant diseases^27^. They consist of several hundred nucleotides and are circular, but hydrogen bonding causes the circles to assume a rod-like structure. We found that their thickness is around 0.6–1.0 nm (Fig. S1). We also deposited synthetic polyadenylic acid molecules on a mica surface and prepared the sample in the same way as those dried just once. The observed thicknesses of the polymers were again 0.6–1.0 nm (Fig. S2). We conclude that the polymers generated by wet-dry cycling match the appearance of known RNA/DNA samples in the AFM^28,29^. However, we cannot conclude that the polymers synthesized by wet-dry cycling are equivalent to biological RNA with respect to their chemical properties. The ribose of a nucleotide monomer has hydroxyl (–OH) groups on the 2′ and 3′ carbon of its ring and therefore can form both 2′–5′ and 3′–5′ ester bonds with the phosphate. Ferris et al.^30^ has used activated AMP in the form of imidazole esters to investigate whether mineral surfaces can catalyze polymerization and observed that montmorillonite can do so. It was reported that both 2′–5′ and 3′–5′ bonds are formed in the non-enzymatic reaction, with 3′–5′ being more abundant. Two RNAse enzymes were used to quantify the ratio, one able to hydrolyze all the bonds and the other only the 3′–5′ bonds. Chromatographic analysis then established the ratio. Currently there is no method in AFM that can determine the type of bonding. Biological RNA synthesis is catalyzed by a polymerase and only 3′–5′ ester bonds are present in the products.

The polymers observed by AFM are also consistent with previous studies in which wet-dry cycling produced oligomers and short polymers that could be detected by nanopore analysis and gel electrophoresis. However, AFM images of polymers several thousand nucleotides in length have not been previously reported. Even though mica is known to be negatively charged in water which might affect the initial binding, we cannot conclude that mica has a special catalytic property which promotes such long products. It is more likely that they are rare and tend to adhere to surfaces and therefore have not been detected by the usual analytical methods such as HPLC, gel electrophoresis or mass spectrometry. During wet-dry cycling on mica surfaces, rare polymers will tend to adsorb to the surface and become concentrated. AFM can then visualize them as single molecules.

In addition to the long polymers we also observed rings consistent with circular RNA. It has long been speculated that circular RNA could be an ancient replicator in the RNA world, since it has the potential to reproduce itself without requiring a protein catalyst^31–33^. The ring structure has another advantage in a replication cycle. The strand inhibition problem^34^ describes how non-enzymatic RNA replication of long polymers is inhibited by the binding between the base pairs. This generates long duplex strands that do not separate quickly enough to allow monomers to undergo complementary base pairing along the single stranded template. Longer templates require even higher temperature ranges to become separated^34,35^. In wet-dry cycles a short single stranded template would therefore quickly grow into a long stable duplex strand with new monomers being added to the ends. This scenario is consistent with the long-tangled structures shown in Fig. 4. The rings are composed of just a few hundred nucleotides and are unable to elongate further. However, they could potentially melt upon rehydration at 80 °C and act as templates during the next dry phase. This scenario is consistent with what we observe in Fig. 5, In which 0.5 nm thick rings have long strands connected to them.

In conclusion, we have confirmed the previously reported synthesis of RNA-like polymers by hot wet dry cycling of mononucleotides and visualized the products as very long polymer tangles or rings. We have verified that covalently bound polymers are not produced at low temperatures, but aggregate structures and irregular linear strings consistent with hydrogen bonded mononucleotides are apparent. We also confirmed the production of ring structures with small polymers connected to the rings and suggest the possibility that these reveal a templating process involving rolling circle synthesis.

The implications of these results lead to a novel origin life scenario. If relatively simple hot wet-dry cycling of mononucleotides has sufficient energy to synthesize polymers thousands of monomers in length, these are sufficiently long to fold into functional ribozymes. However, strand inhibition might make such long polymers too stable for template copying to take place. If RNA rings composed of hundreds of nucleotides are exposed to multiple wet-dry cycles at elevated temperatures, they could undergo transient melting in the hydrated phase followed by template directed synthesis in the dry phase, thereby offering a solution to the strand inhibition problem^27^. It is possible that the first replicating nucleic acid structures were RNA rings able to take the initial steps toward a molecular system capable of Darwinian evolution.

## Methods

### Control: room temperature drying

We added 20–40 µl of the mononucleotide solutions to the surface of freshly cleaved mica which was cut to fit the stage of the AFM. The solution was dried under a gentle stream of pure nitrogen gas. After 30 min the dry mica surface was flushed with 3–5 mL of Milli-Q water and again dried under a stream of nitrogen gas. The sample was immediately mounted and examined in the AFM.

### Control: room temperature wet-dry cycles

We added 20–40 µl of the mononucleotide solutions to the surface of freshly cleaved mica which was dried with nitrogen as described above. The solution was allowed to dry for 30 min, then rehydrated with 20–40 µL ultra-pure deionized water (Milli-Q). This was repeated three times. After the final dry cycle, the mica surface was flushed with Milli-Q water (3–5 mL) and dried under a gentle stream of nitrogen gas. The sample was immediately mounted and examined in the AFM.

### Hot wet dry cycles

We added 20–40 µl of the mononucleotide solutions to the surface of freshly cleaved mica as described above. The mica with nucleotide solution was then placed on a laboratory hot plate maintained at 80 °C. The solution was allowed to dry for 30 min after which the mica surface was rehydrated with 20–40 µL ultra-pure deionized water (Milli-Q). This was repeated three times. After the final dry cycle, the mica surface was flushed with Milli-Q water (3–5 mL) and dried under a gentle stream of nitrogen gas. The sample was immediately mounted and examined in the AFM.

### Coverage

We can estimate how thick the layer of molecules was on the mica when the solutions dried up. Evaporating a drop containing 20–40 µl of a 10 mM solution covering an area of 1 square centimeter results in a 30–60 molecules thick film on average, corresponding roughly to a 10–30 nm thick film. The assumption is that the condensation reactions that synthesize polymers took place within this 10–30 nm dry film. After the wet-dry cycles the molecules that did not participate in polymer synthesis dissolved and were flushed away when the mica was rinsed with water, but some polymers adhered to the surface and remained behind.

### AFM imaging and tip shape

We used a Cypher from Asylum (now Oxford instruments) equipped with a standard AC240 silicon tip from Olympus with a spring constant around 2 nN/nm and a resonant frequency around 70 kHz. Images were at least 512 × 512 pixels. The thickness of the polymers was measured using the section analysis tool in the Igor pro control software for the AFM.

The spatial resolution of the AFM depends on several things. The width of the polymers viewed by AFM depends on a convolution with the tip shape and the polymers. Typically, the tip apex can be described as a half sphere with a radius of curvature of 7 nm for the set of tips used in this study, which is much larger than the 0.5–1 nm thick polymers or the 0.1–0.3 nm thick strings. Using some basic geometrical calculations, a 0.5 nm thick polymer that is cylindrical in shape would appear to be 5 nm wide with a tip with 7 nm radius of curvature. With this type of tip a single phosphate group would appear as a cone with a base diameter of around 4 nm. If two phosphate groups were close to each other they would need to be separated by 1.5 nm for the tip to reach a 0.1 nm height. There is variation in tip shape and the phosphate group and the polymer are not completely spherical or a cylinder, so these numbers can vary accordingly. When measuring the width of the polymers in the AFM they range from 10 to 15 nm. This is wider than the expected 5 nm, however measurements of the width of DNA strands, viroid RNA, or polyadenylic acid recorded with the same type of tip also fall within the range of 10–15 nm. Because the thickness of an isolated polymer on a flat surface is independent of the tip shape, we therefore rely on the thickness rather than the width of the polymers.

### Materials

Sources of mononucleotides: adenosine 5′-monophosphate (Sigma-Aldrich), uridine 5′-monophosphate (P-L Biochemicals), guanosine 5′ monophosphate (Sigma-Aldrich), cytidine 5′-monophosphate (Sigma-Aldrich). We used ultra-pure deionized water (Milli-Q) from a Millipore system to prepare 10 mM solutions of the nucleotides. All solutions were filtered using Pierce™ Protein Concentrators PES, 3K MWCO, 0.5 mL, centrifuged at 14,000 rpm.

Mica was purchased from SPI Supplies (Mica grade v-4). Scotch tape was used to repeatedly cleave the mica until a complete cleavage was achieved after 3–5 times. In addition to the controls described in the text, we ran blind tests with wet-dry cycles using only Milli-Q water to verify the cleaning procedures. This assured that the polymers and rings were not due to an unknown contaminant in the water.

## Supplementary Information

Supplementary Legends.

Supplementary Figure S1.

Supplementary Figure S2.

Supplementary Information 1.

## Data Availability

Copies of the original AFM data will be provided upon reasonable request to Tue Hassenkam (tue@nano.ku.dk or tue.hassenkam@sund.ku.dk).
