# Supplementary material for: Visualizing RNA polymers produced by hot wet-dry cycling
Source: Sci Rep. 2022 Jun 23;12:10098. doi: 10.1038/s41598-022-14238-2 (PMC9226162; doi:10.1038/s41598-022-14238-2)
Supplement: Supplementary file 1 — Supplementary Legends. [file 41598_2022_14238_MOESM1_ESM.docx]

**Supporting data:**

**Figure S1: AFM images of viroids deposited on a mica surface. The cutouts show more than 42 viroids of different shapes and sizes. Most are rod-like in shape, less than 100 nm long, but there were a few open rings, 30-50 nm in diameter as well. The thickness of the rods and rings vary from about 0.6-0.7 nm for the thickest to 0.4-0.5 nm for the thinnest ones.**

**Figure S2: AFM images of polyadenylic acid (polyA) deposited on a mica surface. The sample was created by exposing a mica surface to a solution of polyA for about a minute, before rinsing the still wet surface with pure water. The sample was not wet-dry cycled, so the polymers would therefore have been suspended in solution before adhering to the mica surface. The average thickness of the parts of the polymers that were not curled up was about 0.8 nm. The black bar represents 500 nm.**
