# Supplementary figures and images for: Visualizing RNA polymers produced by hot wet-dry cycling
Source: Sci Rep. 2022 Jun 23;12:10098. doi: 10.1038/s41598-022-14238-2 (PMC9226162; doi:10.1038/s41598-022-14238-2)

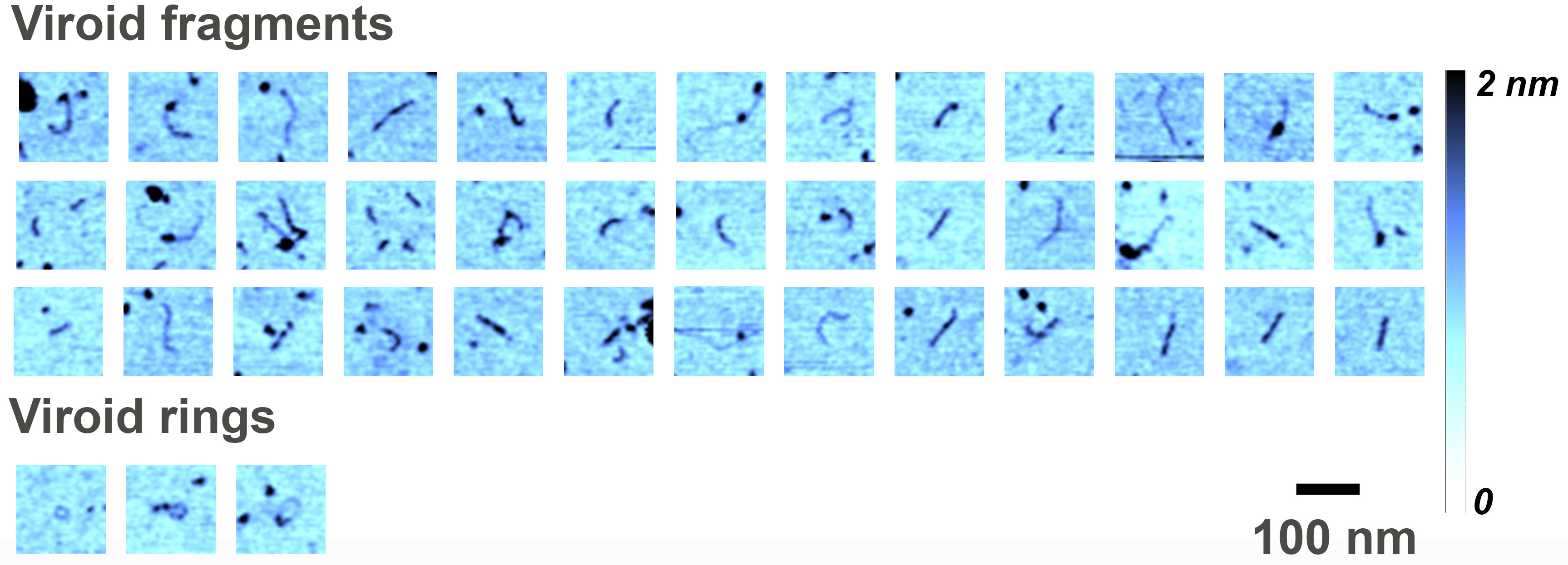

Supplement: Supplementary file 2 — Supplementary Figure S1. [file 41598_2022_14238_MOESM2_ESM.jpg]

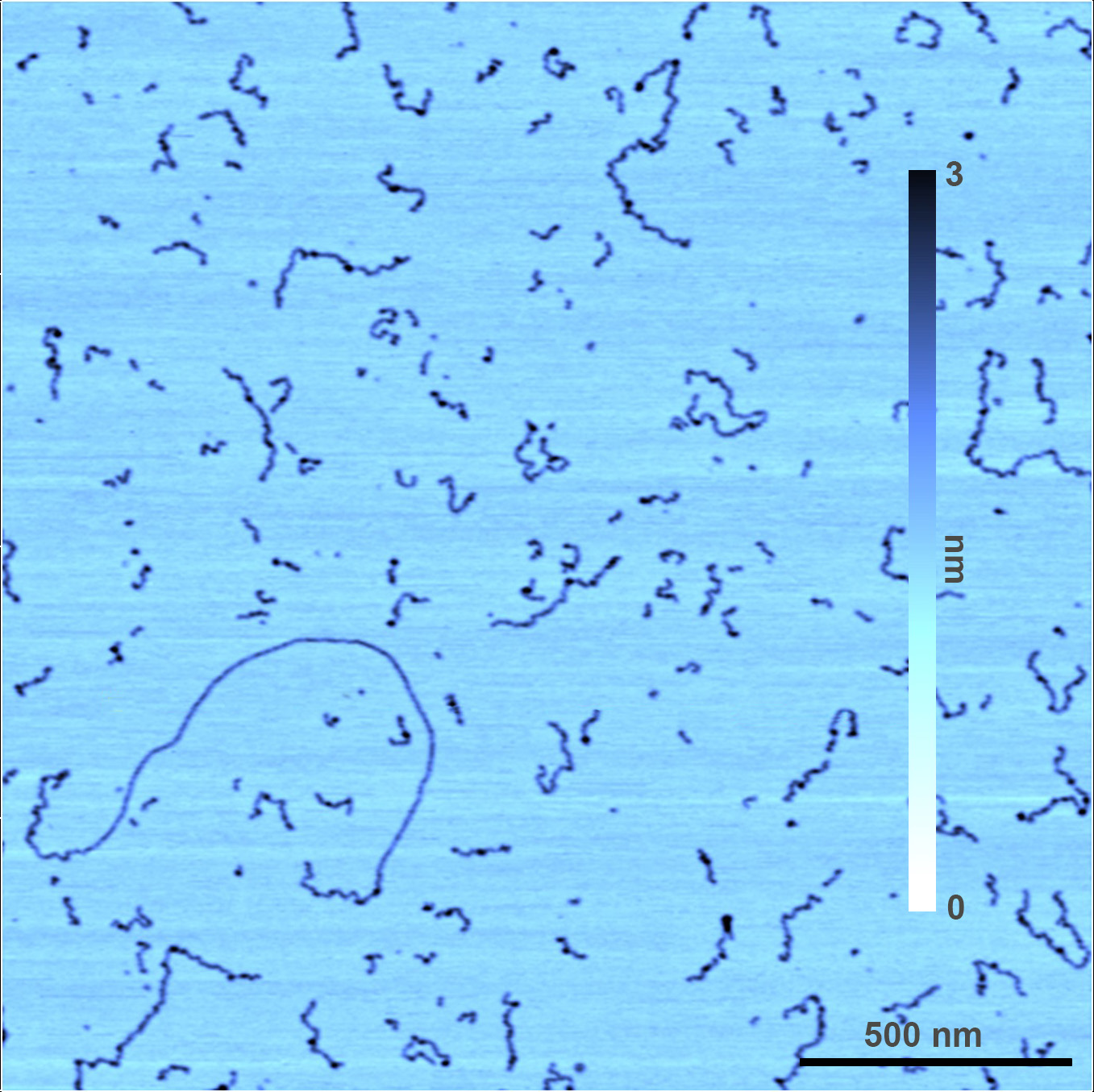

Supplement: Supplementary file 3 — Supplementary Figure S2. [file 41598_2022_14238_MOESM3_ESM.jpg]
