# Supplementary material for: Visualizing RNA polymers produced by hot wet-dry cycling
Source: Sci Rep. 2022 Jun 23;12:10098. doi: 10.1038/s41598-022-14238-2 (PMC9226162; doi:10.1038/s41598-022-14238-2)
Supplement: Supplementary file 4 — Supplementary Information 1. [file 41598_2022_14238_MOESM4_ESM.docx]

# Supplemental note on statistics:

What is the likelihood of getting a false positive. If what we observe are only contamination polymers, we should observe them in the control, in the room temperature experiments and in the hot wet-dry cycle experiments. Here we assume that the probability of observing contamination polymers is the same across the samples. Each AFM image is a random independent sampling of the surface. We therefore define two cases. Either we observe polymers in the AFM image or we do not. We do not worry about the extend or number of polymers in each image, just if they are present or not. We recorded a minimum of 9 images in the controls and observed no polymers in any of these, and we observed polymers in 5 out of 9 AFM images of the hot wet-dry samples. Let’s assume that if there were contamination polymers present they would be covering the surface somewhere between 0 and 100%. Using the binomial distribution (either we observe polymers or we don't in a given AFM image), we can then calculate the likelihood *p*(x) of getting what we observe (0 out of 9 in the controls and 5 out 9 in the Hot wet dry cycles) vs the coverage of contamination polymers:

$$p\left( x \right)=\frac{9!}{5!4!}\left( \frac{x}{9} \right)^{5}\left( \frac{9-x}{9} \right)^{4}\left( \frac{9-x}{9} \right)^{9}$$


Therefore, the likelihood of getting what we observe if the polymers were only contamination would reach a maximum of 0.003 if the assumed coverage of contamination polymers is around 2/9, or we are at least 99,7% confident our result is not just contamination polymers.
